# Supplementary material for: A comparison of reference-based algorithms for correcting cell-type heterogeneity in Epigenome-Wide Association Studies
Source: BMC Bioinformatics. 2017 Feb 13;18:105. doi: 10.1186/s12859-017-1511-5 (PMC5307731; doi:10.1186/s12859-017-1511-5)
Supplement: Additional file 1: — Contains all Supplementary Figures and Supplementary Tables plus their captions: Figure S1. Clustering validation of reference blood database. Figure S2. Validation of EpiDISH. Figure S3. Improvement of inference using DHS data in 3 independent data sets. Figure S4. Comparison of reference-based methods. Figure S5. Comparative performance of reference-based methods in an EWAS of smoking. Table S1. The blood reference database. Table S2. Gold-standard list of 62 smoking-associated DMCs (sDMCs). Table S3. Smoking-associated DMCs with no adjustment for cell-type composition. Table S4. Smoking-associated DMCs as obtained using EpiDISH. (PDF 1241 kb) [file 12859_2017_1511_MOESM1_ESM.pdf]

SUPPLEMENTARY INFORMATION DOCUMENT

FOR

A comparison of reference-based algorithms for correcting cell-type heterogeneity in Epigenome-Wide Association Studies

Andrew E. Teschendorff [1,2,3,*], Charles E. Breeze [4], Shijie C Zheng [1,5] and Stephan Beck [4]

Corresponding author: Andrew E. Teschendorff- a.teschendorff@ucl.ac.uk

1. CAS Key Lab of Computational Biology, CAS-MPG Partner Institute for Computational Biology, Shanghai Institute for Biological Sciences, Chinese Academy of Sciences, Shanghai 20031, China.
2. Department of Women's Cancer, University College London, 74 Huntley Street, London WC1E 6AU, United Kingdom.
3. Statistical Cancer Genomics, Paul O'Gorman Building, UCL Cancer Institute, University College London, 72 Huntley Street, London WC1E 6BT, United Kingdom.
4. Medical Genomics, Paul O'Gorman Building, UCL Cancer Institute, University College London, 72 Huntley Street, London WC1E 6BT, United Kingdom.
5. University of Chinese Academy of Sciences, 19A Yuquan Road, Beijing 100049, China.

DESCRIPTION:
This document contains all Supplementary Tables and Supplementary Figures.

SUPPLEMENTARY FIGURES

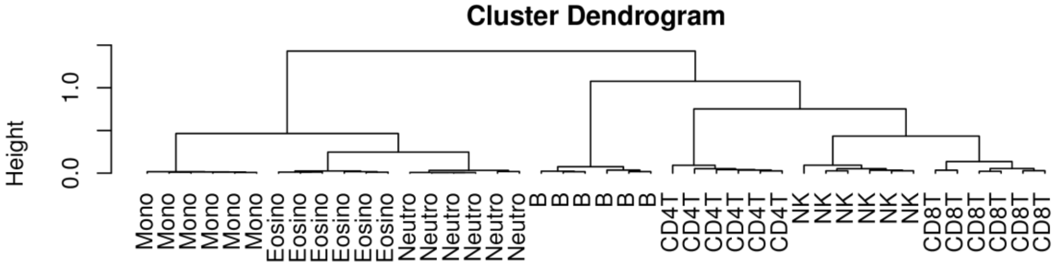

fig.S1: Clustering validation of reference blood database. Clustering of purified blood cell subtypes from Reinius et al [1] over the 333 CpGs making up the reference blood database.

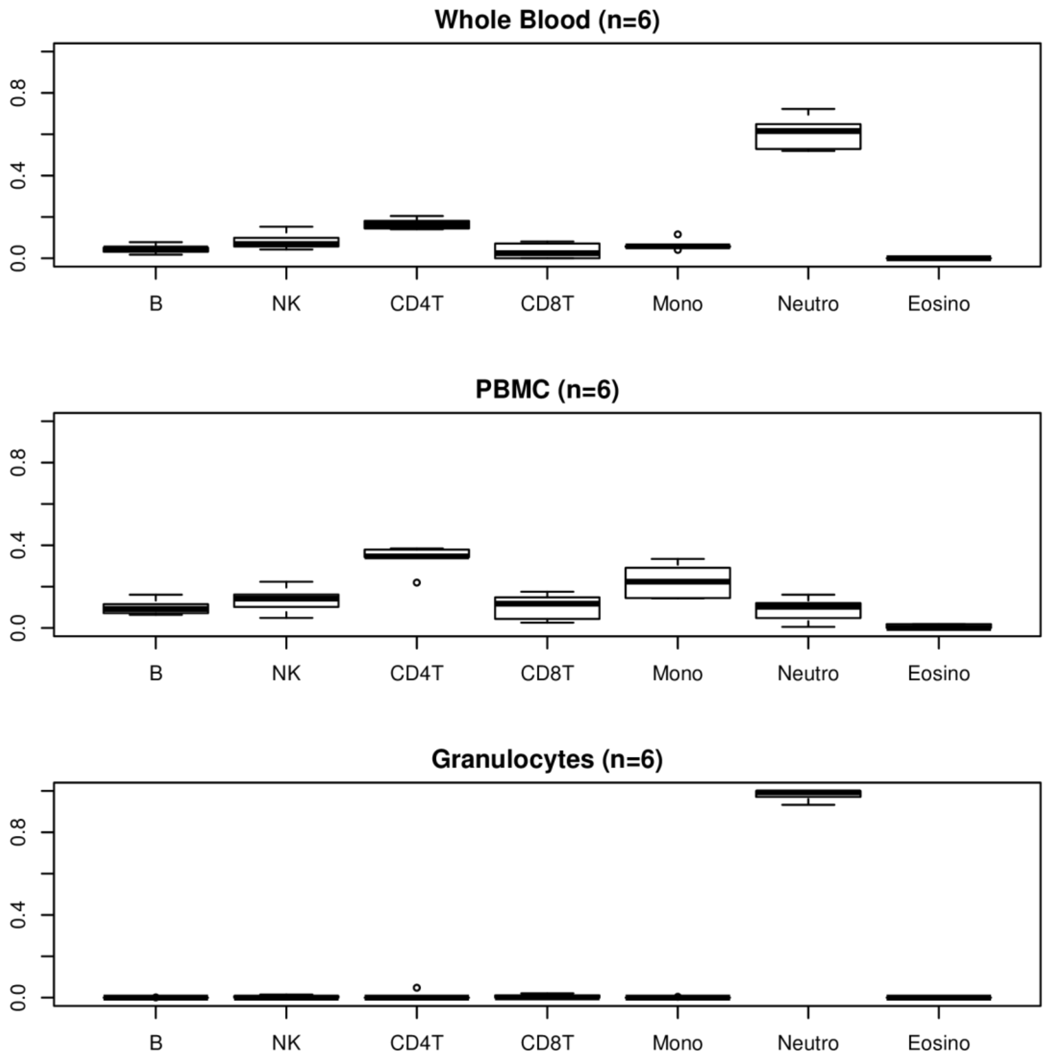

fig.S2: **Validation of EpiDISH.** EpiDISH estimates of the cell subtype proportions (y-axis) of 6 whole blood samples, 6 peripheral blood mononuclear cell samples and 6 granulocyte samples. These samples were taken from Reinius et al, the same dataset from which the purified blood cell types used in the reference centroid construction came from. B=B-cell, NK=Natural Killer, CD4T=CD4+ T-cells, CD8T=CD8+ T-cells, Mon=Monocytes, Neu=Neutrophils, Eos=Eosinophils).

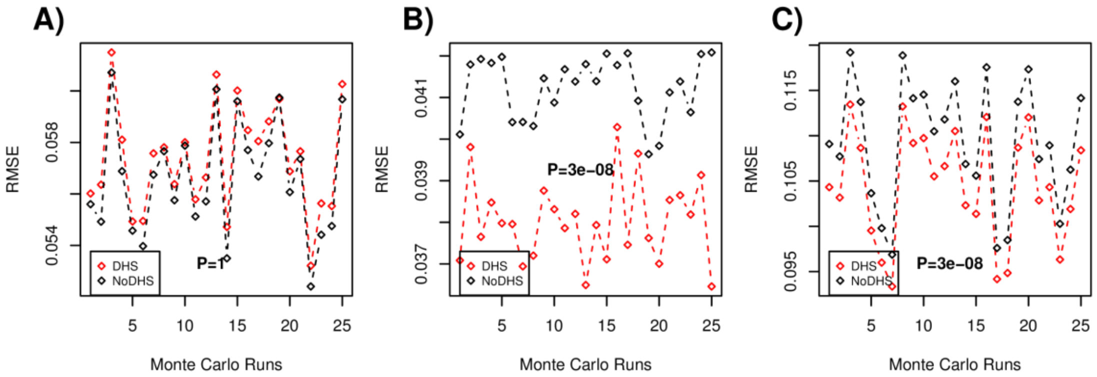

fig.S3: Improvement of inference using DHS data in 3 independent data sets. A) Using 100 in-silico mixtures of 5 purified blood cell subtypes from Zilbauer et al [2], we compare the Root Mean Square Error values (RMSE, y-axis) of the estimated cell proportions as obtained using EpiDISH between the two different reference databases: one which uses cell-type specific DHSs to select DMCs when constructing the DNAm reference centroids (DHS), and another which only uses DMCs regardless of DHS status (noDHS). The average RMSE value over all cell-types is being shown. A total of 25 different Monte Carlo runs were performed to obtain 25 average RMSE values (x-axis) for each reference database. P-value is from a one-tailed paired Wilcoxon-rank sum test. **B)** As A) but now 100 in-silico generated mixtures of 3 epithelial cell subtypes (breast, renal cortical and pancreas) for which DHS data was available**.** **C)** As B), but now for 3 other cell subtypes (fetal lung fibroblast-IMR90, hepatocytes and B-cells) with available DHS information and DNAm profiles from two independent studies.

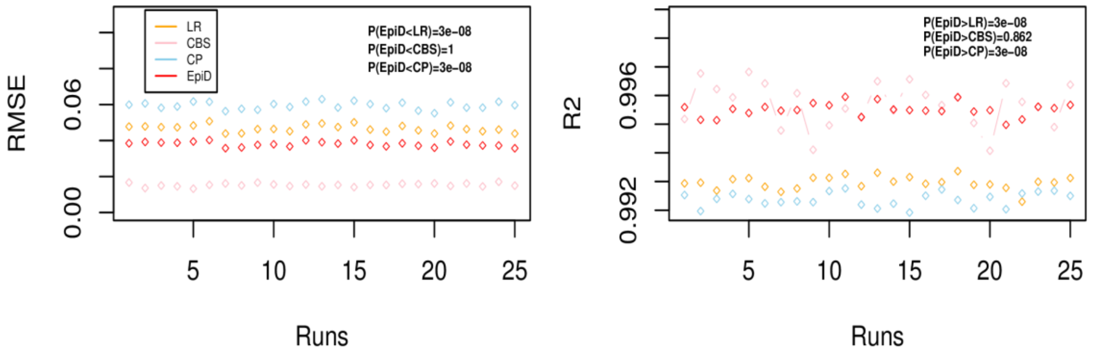

fig.S4: Comparison of reference-based methods. Left panel: RMSE values of EpiDISH and those of the 3 other reference-based algorithms (LR=linear multivariate regression, CP=constrained projection and CIBERSORT=CBS), as obtained from over 100 in-silico randomly generated mixtures of 3 epithelial cell subtypes (human mammary epithelial cells, human renal cortical epithelial cells and human pancreas). A total of 25 Monte-Carlo runs were performed. P-values are shown between EpiDISH and all others, and are derived from a one-tailed paired Wilcoxon rank sum test. **Right panel:** as left panel, but for the R^2 values.

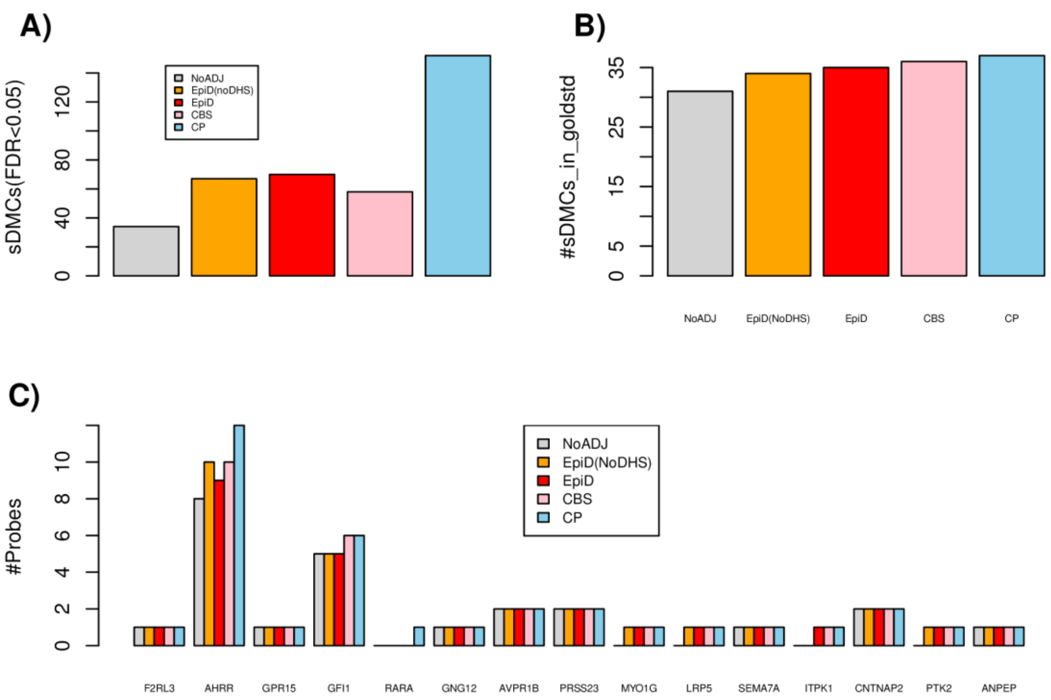

fig.S5: Comparative performance of reference-based methods in an EWAS of smoking. A) Comparison of the number of smoking-associated DMCs (sDMCs) at FDR<0.05 for each of 5 methods: NoADJ=no-adjustment, EpiD(noDHS)=adjustment with RPC using reference with no DHS info, EpiD=adjustment with RPC and a reference with DHS info, CBS=adjustment with CIBERSORT, CP=adjustment with constrained projection. **B)** Comparison of the number of sDMCs identified by each method that mapped to an independently generated gold-standard list of 62 smoking-associated CpGs from Gao et al [3]. **C)** Among the sDMCs identified in A), we indicate the numbers of these that map to the 15 unique genes implicated in the gold-standard list of 62 smoking-associated CpGs.

SUPPLEMENTARY TABLES

probeID	B	NK	CD4T	CD8T	Gran	Mono	Neutro	Eosino
cg01024458	0.034	0.962	0.969	0.958	0.994	0.987	0.992	0.991
cg11661493	0.037	0.954	0.971	0.964	0.961	0.958	0.962	0.962
cg21596498	0.06	0.98	0.989	0.989	0.978	0.98	0.976	0.984
cg05205074	0.049	0.954	0.967	0.961	0.981	0.967	0.979	0.986
cg17232476	0.045	0.962	0.969	0.958	0.976	0.932	0.98	0.976
cg14936008	0.065	0.976	0.98	0.983	0.981	0.977	0.983	0.986

cg05579731	0.041	0.94	0.953	0.948	0.964	0.969	0.966	0.961
cg17995557	0.04	0.87	0.974	0.958	0.976	0.962	0.969	0.968
cg15035590	0.072	0.978	0.986	0.979	0.984	0.974	0.986	0.986
cg03248292	0.021	0.917	0.956	0.919	0.941	0.925	0.927	0.918
cg00760938	0.049	0.921	0.964	0.946	0.95	0.945	0.949	0.964
cg21743182	0.054	0.941	0.955	0.955	0.957	0.944	0.959	0.955
cg26552743	0.045	0.936	0.964	0.963	0.941	0.932	0.932	0.922
cg22212560	0.043	0.966	0.978	0.976	0.968	0.821	0.956	0.934
cg20602300	0.086	0.974	0.983	0.978	0.98	0.978	0.977	0.983
cg06889975	0.067	0.961	0.979	0.966	0.937	0.952	0.945	0.936
cg15532942	0.063	0.928	0.956	0.937	0.963	0.963	0.958	0.97
cg02665297	0.08	0.957	0.97	0.969	0.974	0.965	0.972	0.975
cg16148346	0.072	0.965	0.969	0.96	0.968	0.943	0.959	0.962
cg22498365	0.074	0.961	0.942	0.955	0.974	0.962	0.97	0.977
cg27565966	0.068	0.938	0.967	0.947	0.949	0.942	0.943	0.957
cg18184053	0.022	0.81	0.957	0.88	0.934	0.916	0.917	0.92
cg26810157	0.044	0.891	0.974	0.943	0.958	0.943	0.92	0.858
cg24466100	0.044	0.93	0.916	0.897	0.92	0.911	0.933	0.941
cg07597976	0.023	0.867	0.947	0.945	0.898	0.88	0.88	0.88
cg22907103	0.056	0.932	0.952	0.944	0.942	0.9	0.934	0.936
cg12976793	0.106	0.973	0.977	0.973	0.985	0.984	0.98	0.981
cg14060402	0.097	0.971	0.977	0.978	0.969	0.956	0.967	0.965
cg26795340	0.049	0.917	0.948	0.927	0.921	0.904	0.918	0.909
cg15287850	0.058	0.932	0.952	0.939	0.951	0.924	0.927	0.902
cg09469986	0.087	0.922	0.966	0.941	0.972	0.961	0.977	0.97
cg04828493	0.078	0.891	0.97	0.963	0.984	0.892	0.981	0.981
cg10147394	0.059	0.895	0.926	0.89	0.953	0.946	0.952	0.944
cg03980424	0.108	0.971	0.943	0.985	0.988	0.966	0.984	0.983
cg16185947	0.061	0.939	0.967	0.94	0.9	0.936	0.905	0.843
cg02582213	0.1	0.954	0.964	0.957	0.967	0.948	0.967	0.969
cg18067520	0.027	0.932	0.967	0.974	0.897	0.828	0.879	0.737
cg18590995	0.041	0.929	0.943	0.946	0.873	0.887	0.848	0.85
cg08939052	0.066	0.868	0.94	0.888	0.958	0.942	0.952	0.962
cg25131632	0.072	0.88	0.936	0.887	0.963	0.955	0.959	0.965
cg13915752	0.088	0.961	0.971	0.956	0.96	0.928	0.948	0.912
cg26160564	0.064	0.86	0.918	0.879	0.958	0.938	0.96	0.964
cg01869288	0.039	0.799	0.915	0.787	0.951	0.936	0.95	0.961
cg18664915	0.089	0.944	0.947	0.932	0.945	0.934	0.932	0.949
cg06963205	0.041	0.882	0.902	0.883	0.939	0.834	0.928	0.921
cg23730277	0.117	0.953	0.98	0.971	0.973	0.969	0.959	0.956
cg11160673	0.057	0.935	0.964	0.98	0.918	0.691	0.933	0.922
cg11193201	0.051	0.945	0.943	0.951	0.915	0.819	0.91	0.813
cg00226923	0.08	0.884	0.94	0.902	0.942	0.934	0.946	0.942
cg20666585	0.072	0.823	0.951	0.864	0.959	0.94	0.956	0.962

cg27582527	0.895	0.07	0.903	0.427	0.966	0.969	0.972	0.975
cg06202778	0.98	0.149	0.957	0.555	0.956	0.932	0.965	0.969
cg09345868	0.97	0.144	0.789	0.417	0.977	0.978	0.986	0.986
cg26934362	0.85	0.109	0.851	0.329	0.941	0.956	0.949	0.966
cg05653887	0.959	0.172	0.935	0.507	0.939	0.959	0.947	0.948
cg07307830	0.948	0.151	0.76	0.487	0.963	0.963	0.968	0.978
cg21634628	0.816	0.165	0.87	0.532	0.971	0.969	0.971	0.979
cg02384859	0.931	0.179	0.926	0.494	0.959	0.956	0.95	0.967
cg03708221	0.905	0.154	0.878	0.482	0.913	0.934	0.927	0.936
cg02150910	0.813	0.166	0.925	0.502	0.952	0.96	0.951	0.963
cg20657709	0.97	0.225	0.968	0.625	0.967	0.968	0.96	0.968
cg03538296	0.979	0.256	0.983	0.737	0.983	0.983	0.985	0.979
cg19696012	0.946	0.189	0.881	0.518	0.95	0.972	0.965	0.957
cg27309871	0.953	0.229	0.966	0.614	0.954	0.965	0.97	0.97
cg21474838	0.976	0.221	0.885	0.588	0.971	0.979	0.972	0.978
cg05875239	0.932	0.241	0.946	0.739	0.947	0.933	0.949	0.969
cg24854175	0.945	0.143	0.743	0.331	0.944	0.967	0.944	0.952
cg01940139	0.975	0.228	0.91	0.59	0.975	0.974	0.966	0.975
cg14301562	0.935	0.193	0.936	0.577	0.885	0.932	0.894	0.9
cg18672716	0.926	0.167	0.907	0.467	0.895	0.943	0.891	0.878
cg26227465	0.875	0.153	0.756	0.451	0.934	0.928	0.939	0.944
cg13613174	0.959	0.162	0.789	0.394	0.855	0.956	0.888	0.957
cg09261289	0.974	0.268	0.972	0.693	0.975	0.978	0.973	0.984
cg15241779	0.936	0.249	0.945	0.667	0.96	0.968	0.966	0.969
cg20460771	0.933	0.177	0.874	0.465	0.904	0.91	0.909	0.908
cg05224770	0.74	0.108	0.694	0.35	0.948	0.919	0.938	0.934
cg13617280	0.936	0.247	0.929	0.833	0.862	0.948	0.845	0.918
cg04780380	0.829	0.116	0.692	0.273	0.936	0.944	0.93	0.945
cg10628126	0.952	0.232	0.875	0.643	0.975	0.865	0.975	0.985
cg13828440	0.791	0.223	0.934	0.695	0.946	0.935	0.936	0.944
cg12996622	0.831	0.175	0.859	0.528	0.888	0.899	0.892	0.892
cg26012482	0.93	0.218	0.817	0.55	0.952	0.97	0.951	0.924
cg14384416	0.947	0.238	0.928	0.526	0.952	0.944	0.945	0.969
cg10142452	0.899	0.228	0.895	0.572	0.931	0.943	0.944	0.945
cg22749642	0.861	0.205	0.911	0.506	0.901	0.936	0.924	0.912
cg18310515	0.952	0.243	0.89	0.57	0.936	0.949	0.947	0.955
cg17766305	0.249	0.898	0.516	0.749	0.076	0.023	0.059	0.015
cg18461635	0.916	0.184	0.692	0.429	0.945	0.946	0.944	0.95
cg21333217	0.98	0.287	0.96	0.6	0.967	0.971	0.972	0.979
cg02663352	0.562	0.182	0.881	0.52	0.954	0.941	0.956	0.965
cg07065759	0.003	0.784	0.344	0.62	0.015	0.007	0.015	0.002
cg11335172	0.987	0.324	0.975	0.749	0.971	0.988	0.978	0.975
cg16212145	0.907	0.248	0.898	0.551	0.948	0.949	0.949	0.935
cg20959189	0.825	0.169	0.714	0.291	0.953	0.973	0.954	0.956

cg18748064	0.863	0.233	0.877	0.477	0.955	0.963	0.956	0.958
cg02902412	0.971	0.267	0.982	0.441	0.961	0.969	0.952	0.978
cg19828970	0.023	0.929	0.854	0.928	0.04	0.03	0.04	0.02
cg18408209	0.897	0.239	0.915	0.506	0.933	0.936	0.919	0.931
cg01726890	0.923	0.195	0.852	0.422	0.883	0.896	0.883	0.861
cg15036529	0.793	0.186	0.611	0.475	0.962	0.974	0.94	0.968
cg00366435	0.978	0.939	0.15	0.632	0.979	0.984	0.963	0.93
cg13468685	0.15	0.076	0.895	0.593	0.045	0.039	0.053	0.021
cg24462702	0.933	0.816	0.132	0.838	0.848	0.879	0.865	0.847
cg26166854	0.973	0.773	0.131	0.478	0.97	0.979	0.961	0.987
cg04712122	0.96	0.826	0.1	0.196	0.927	0.968	0.959	0.987
cg06915826	0.973	0.895	0.147	0.408	0.955	0.96	0.963	0.978
cg11848483	0.971	0.917	0.153	0.371	0.981	0.982	0.969	0.991
cg07679948	0.968	0.89	0.163	0.474	0.955	0.969	0.951	0.984
cg02380585	0.01	0.268	0.903	0.886	0.032	0.009	0.037	0.002
cg12465678	0.425	0.093	0.908	0.622	0.055	0.032	0.058	0.018
cg01874152	0.893	0.888	0.168	0.482	0.98	0.983	0.976	0.988
cg23723835	0.717	0.891	0.136	0.489	0.963	0.972	0.965	0.98
cg07713946	0.975	0.831	0.155	0.398	0.968	0.978	0.963	0.977
cg10240150	0.96	0.811	0.141	0.392	0.938	0.953	0.942	0.967
cg12101498	0.02	0.118	0.85	0.623	0.111	0.067	0.091	0.017
cg09885622	0.214	0.118	0.915	0.664	0.213	0.127	0.181	0.041
cg02661764	0.908	0.905	0.163	0.533	0.877	0.848	0.898	0.942
cg24706505	0.229	0.106	0.837	0.534	0.04	0.056	0.044	0.014
cg07837085	0.237	0.055	0.794	0.391	0.037	0.038	0.041	0.015
cg07545925	0.952	0.687	0.106	0.068	0.963	0.973	0.956	0.982
cg19628988	0.173	0.444	0.933	0.907	0.091	0.019	0.051	0.025
cg10782923	0.944	0.886	0.198	0.498	0.92	0.929	0.931	0.971
cg13651908	0.953	0.813	0.179	0.461	0.947	0.952	0.922	0.938
cg16452866	0.969	0.784	0.156	0.227	0.953	0.978	0.957	0.977
cg01987702	0.018	0.108	0.761	0.463	0.02	0.016	0.014	0.002
cg11944101	0.902	0.85	0.096	0.852	0.621	0.683	0.625	0.595
cg04244970	0.247	0.033	0.759	0.292	0.023	0.016	0.033	0.009
cg24527636	0.9	0.782	0.122	0.191	0.91	0.908	0.906	0.958
cg05074138	0.953	0.801	0.181	0.406	0.931	0.943	0.932	0.941
cg08884752	0.859	0.881	0.142	0.48	0.855	0.812	0.851	0.839
cg27207932	0.96	0.878	0.082	0.806	0.378	0.715	0.432	0.57
cg12353788	0.954	0.787	0.164	0.357	0.889	0.935	0.904	0.911
cg20661080	0.925	0.809	0.192	0.395	0.961	0.961	0.958	0.963
cg09662852	0.085	0.066	0.76	0.463	0.042	0.03	0.046	0.017
cg25416125	0.914	0.819	0.171	0.432	0.892	0.901	0.89	0.924
cg04776231	0.129	0.33	0.877	0.877	0.066	0.022	0.048	0.01
cg13606287	0.974	0.567	0.168	0.382	0.972	0.978	0.966	0.987
cg21400549	0.485	0.33	0.927	0.735	0.113	0.064	0.091	0.009

cg25226014	0.96	0.582	0.152	0.292	0.972	0.974	0.966	0.982
cg10099758	0.048	0.175	0.813	0.613	0.135	0.045	0.138	0.019
cg10837404	0.988	0.962	0.321	0.838	0.983	0.988	0.987	0.992
cg14564351	0.123	0.156	0.746	0.309	0.056	0.029	0.035	0.006
cg16193207	0.972	0.897	0.245	0.508	0.969	0.969	0.959	0.975
cg24902461	0.095	0.518	0.9	0.849	0.073	0.04	0.067	0.023
cg10599446	0.958	0.658	0.146	0.269	0.9	0.926	0.938	0.929
cg07015803	0.957	0.742	0.167	0.234	0.934	0.963	0.935	0.97
cg18154117	0.866	0.816	0.176	0.45	0.909	0.892	0.899	0.921
cg11230435	0.05	0.028	0.721	0.408	0.014	0.025	0.024	0.003
cg04642300	0.18	0.042	0.758	0.475	0.025	0.023	0.034	0.013
cg07930673	0.954	0.701	0.145	0.12	0.949	0.957	0.948	0.967
cg25939861	0.936	0.61	0.906	0.079	0.948	0.932	0.947	0.961
cg00219921	0.981	0.83	0.915	0.165	0.971	0.968	0.966	0.978
cg10977115	0.971	0.281	0.427	0.023	0.969	0.972	0.96	0.975
cg22512531	0.97	0.248	0.571	0.05	0.965	0.969	0.963	0.978
cg06164961	0.98	0.575	0.137	0.057	0.968	0.976	0.958	0.98
cg26848126	0.024	0.432	0.411	0.871	0.049	0.024	0.055	0.016
cg24456340	0.985	0.262	0.696	0.108	0.972	0.979	0.978	0.933
cg25643644	0.963	0.572	0.122	0.059	0.944	0.956	0.939	0.957
cg20894640	0.97	0.207	0.265	0.036	0.961	0.964	0.95	0.975
cg24841244	0.97	0.668	0.152	0.092	0.949	0.96	0.937	0.971
cg27470208	0.953	0.288	0.19	0.033	0.949	0.953	0.95	0.968
cg22689323	0.973	0.309	0.175	0.036	0.946	0.965	0.945	0.937
cg24612198	0.973	0.598	0.146	0.09	0.965	0.969	0.955	0.979
cg09088496	0.96	0.249	0.171	0.031	0.941	0.959	0.938	0.977
cg07661835	0.962	0.696	0.471	0.153	0.915	0.93	0.939	0.97
cg05356800	0.859	0.25	0.796	0.098	0.89	0.891	0.916	0.927
cg25711558	0.057	0.568	0.873	0.934	0.059	0.021	0.052	0.008
cg13750061	0.956	0.728	0.156	0.116	0.953	0.952	0.958	0.971
cg09453312	0.955	0.123	0.096	0.008	0.945	0.96	0.959	0.97
cg14506192	0.962	0.244	0.558	0.106	0.956	0.958	0.944	0.979
cg09032544	0.956	0.283	0.228	0.05	0.899	0.951	0.916	0.976
cg00467547	0.957	0.154	0.089	0.012	0.96	0.955	0.946	0.971
cg00277591	0.979	0.198	0.138	0.016	0.896	0.956	0.897	0.93
cg27008565	0.933	0.158	0.439	0.061	0.954	0.945	0.937	0.954
cg09354050	0.958	0.3	0.113	0.032	0.927	0.951	0.919	0.923
cg17477578	0.885	0.178	0.149	0.021	0.951	0.962	0.955	0.965
cg11531557	0.935	0.629	0.503	0.157	0.933	0.942	0.937	0.951
cg02324835	0.925	0.646	0.381	0.143	0.937	0.94	0.947	0.963
cg15935770	0.036	0.89	0.939	0.985	0.068	0.018	0.071	0.007
cg22999502	0.824	0.523	0.336	0.086	0.926	0.933	0.903	0.935
cg23318020	0.864	0.555	0.153	0.078	0.933	0.949	0.924	0.959
cg09145126	0.069	0.771	0.901	0.964	0.079	0.027	0.068	0.011

cg22742001	0.054	0.894	0.947	0.98	0.035	0.014	0.032	0.002
cg25025181	0.152	0.661	0.922	0.954	0.033	0.016	0.036	0.001
cg21330949	0.887	0.145	0.219	0.017	0.924	0.933	0.902	0.943
cg09909069	0.962	0.285	0.136	0.049	0.952	0.955	0.939	0.943
cg27111890	0.975	0.668	0.189	0.124	0.937	0.965	0.942	0.922
cg24448340	0.061	0.913	0.932	0.983	0.045	0.022	0.047	0.005
cg11760500	0.84	0.187	0.117	0.023	0.976	0.979	0.956	0.961
cg18463607	0.928	0.299	0.133	0.063	0.967	0.969	0.958	0.985
cg16093065	0.978	0.353	0.177	0.09	0.969	0.972	0.969	0.981
cg09232358	0.949	0.671	0.206	0.128	0.933	0.927	0.931	0.969
cg14278300	0.929	0.259	0.227	0.051	0.909	0.916	0.913	0.945
cg23671196	0.096	0.778	0.797	0.944	0.067	0.035	0.065	0.01
cg20487608	0.012	0.783	0.877	0.934	0.025	0.017	0.027	0.006
cg19994968	0.955	0.643	0.158	0.124	0.949	0.963	0.946	0.959
cg15046489	0.949	0.189	0.252	0.064	0.95	0.956	0.951	0.961
cg26872907	0.963	0.865	0.837	0.963	0.964	0.095	0.957	0.964
cg00701951	0.981	0.922	0.935	0.958	0.754	0.099	0.78	0.941
cg19301114	0.884	0.798	0.852	0.909	0.929	0.068	0.93	0.949
cg26538782	0.741	0.965	0.966	0.989	0.957	0.116	0.948	0.979
cg23244761	0.98	0.911	0.902	0.985	0.982	0.141	0.976	0.982
cg07002540	0.96	0.89	0.886	0.958	0.802	0.115	0.833	0.927
cg08538581	0.67	0.947	0.929	0.962	0.736	0.073	0.765	0.918
cg09220326	0.975	0.936	0.931	0.975	0.455	0.061	0.504	0.777
cg06489037	0.978	0.936	0.927	0.975	0.4	0.073	0.486	0.86
cg18066690	0.977	0.961	0.941	0.978	0.982	0.184	0.971	0.978
cg06297318	0.949	0.944	0.937	0.957	0.906	0.149	0.865	0.932
cg17594003	0.93	0.939	0.959	0.98	0.524	0.073	0.566	0.745
cg16429499	0.411	0.916	0.929	0.977	0.964	0.081	0.966	0.968
cg13430807	0.881	0.862	0.865	0.957	0.93	0.129	0.919	0.927
cg23146741	0.768	0.774	0.865	0.894	0.895	0.089	0.911	0.956
cg17660833	0.952	0.901	0.928	0.948	0.43	0.095	0.546	0.928
cg22957691	0.989	0.889	0.953	0.981	0.081	0.031	0.249	0.744
cg10278149	0.941	0.922	0.938	0.969	0.75	0.116	0.76	0.776
cg27059140	0.968	0.93	0.954	0.968	0.485	0.077	0.513	0.739
cg13093111	0.962	0.924	0.966	0.982	0.502	0.062	0.51	0.624
cg00091349	0.847	0.839	0.869	0.923	0.484	0.031	0.54	0.742
cg10933959	0.986	0.966	0.959	0.99	0.292	0.08	0.395	0.744
cg12207930	0.898	0.913	0.897	0.947	0.657	0.11	0.681	0.887
cg11814087	0.945	0.89	0.922	0.962	0.238	0.059	0.38	0.808
cg09322573	0.903	0.926	0.948	0.976	0.667	0.104	0.663	0.757
cg10624395	0.956	0.931	0.925	0.965	0.981	0.196	0.976	0.963
cg00208012	0.882	0.903	0.894	0.954	0.948	0.163	0.92	0.96
cg12655112	0.878	0.889	0.852	0.956	0.673	0.092	0.689	0.817
cg02190353	0.948	0.894	0.906	0.962	0.12	0.032	0.28	0.726

cg13932501	0.962	0.927	0.926	0.963	0.292	0.064	0.396	0.724
cg03533256	0.895	0.726	0.804	0.806	0.77	0.074	0.794	0.926
cg14985891	0.973	0.917	0.934	0.967	0.088	0.026	0.204	0.653
cg11118440	0.885	0.881	0.829	0.914	0.429	0.078	0.537	0.918
cg08908131	0.912	0.783	0.871	0.87	0.948	0.138	0.937	0.949
cg23391288	0.918	0.929	0.903	0.972	0.987	0.201	0.982	0.99
cg15835339	0.958	0.907	0.927	0.95	0.279	0.069	0.39	0.771
cg19082496	0.963	0.849	0.905	0.914	0.205	0.033	0.312	0.741
cg21683390	0.845	0.905	0.946	0.979	0.385	0.075	0.466	0.798
cg21814550	0.962	0.946	0.951	0.961	0.182	0.059	0.296	0.705
cg04605532	0.97	0.944	0.934	0.983	0.204	0.06	0.305	0.677
cg18990407	0.569	0.925	0.923	0.964	0.869	0.121	0.863	0.929
cg03002688	0.873	0.9	0.824	0.948	0.287	0.05	0.408	0.787
cg02780988	0.772	0.851	0.878	0.902	0.922	0.137	0.918	0.933
cg23363263	0.945	0.96	0.962	0.983	0.178	0.072	0.314	0.692
cg01110616	0.981	0.922	0.959	0.945	0.8	0.178	0.798	0.875
cg20978247	0.94	0.927	0.935	0.984	0.445	0.113	0.502	0.801
cg09310966	0.972	0.855	0.878	0.935	0.474	0.087	0.534	0.757
cg19358608	0.955	0.913	0.922	0.954	0.558	0.098	0.59	0.665
cg05242065	0.933	0.95	0.81	0.968	0.891	0.187	0.917	0.948
cg17541002	0.725	0.93	0.906	0.978	0.153	0.055	0.302	0.892
cg22381196	0.992	0.99	0.99	0.996	0.049	0.815	0.079	0.072
cg21685770	0.94	0.952	0.974	0.976	0.046	0.786	0.079	0.204
cg00439981	0.994	0.979	0.984	0.995	0.046	0.624	0.074	0.008
cg11557901	0.99	0.979	0.974	0.979	0.051	0.472	0.055	0.009
cg05344747	0.819	0.975	0.972	0.987	0.11	0.855	0.094	0.023
cg09859659	0.952	0.948	0.979	0.977	0.133	0.916	0.155	0.192
cg25968394	0.971	0.977	0.976	0.983	0.112	0.721	0.13	0.181
cg04609694	0.976	0.972	0.979	0.978	0.093	0.881	0.209	0.493
cg13468144	0.985	0.978	0.951	0.976	0.112	0.952	0.278	0.832
cg13178361	0.955	0.947	0.958	0.965	0.099	0.759	0.111	0.07
cg13396713	0.983	0.97	0.965	0.985	0.066	0.477	0.071	0.01
cg02021919	0.972	0.96	0.961	0.961	0.105	0.787	0.14	0.157
cg11922563	0.956	0.895	0.931	0.946	0.024	0.386	0.038	0.019
cg25025545	0.964	0.942	0.953	0.974	0.045	0.377	0.055	0.009
cg27200257	0.951	0.933	0.937	0.922	0.086	0.699	0.1	0.02
cg03406844	0.927	0.934	0.934	0.929	0.122	0.828	0.131	0.091
cg09993145	0.119	0.025	0.037	0.009	0.829	0.083	0.83	0.888
cg11354682	0.012	0.053	0.34	0.347	0.933	0.08	0.931	0.945
cg23819411	0.958	0.938	0.95	0.948	0.107	0.68	0.226	0.683
cg16125725	0.979	0.97	0.971	0.979	0.096	0.597	0.244	0.757
cg14059339	0.972	0.953	0.937	0.975	0.064	0.371	0.087	0.098
cg09109411	0.98	0.934	0.959	0.972	0.096	0.431	0.084	0.007
cg05486872	0.968	0.466	0.953	0.803	0.052	0.869	0.049	0.005

cg21341487	0.927	0.91	0.93	0.962	0.072	0.506	0.082	0.015
cg27545615	0.87	0.856	0.829	0.876	0.037	0.625	0.051	0.007
cg13633625	0.93	0.964	0.977	0.974	0.103	0.686	0.254	0.741
cg11597277	0.988	0.977	0.985	0.985	0.177	0.714	0.223	0.424
cg11389756	0.966	0.927	0.937	0.961	0.048	0.247	0.056	0.018
cg07356342	0.964	0.93	0.935	0.957	0.08	0.482	0.135	0.262
cg15033269	0.887	0.895	0.943	0.948	0.06	0.473	0.075	0.015
cg25757820	0.987	0.976	0.99	0.989	0.168	0.836	0.323	0.861
cg25605731	0.872	0.945	0.957	0.954	0.067	0.497	0.091	0.02
cg26341831	0.015	0.046	0.157	0.019	0.873	0.264	0.777	0.467
cg00405190	0.953	0.936	0.887	0.948	0.074	0.465	0.098	0.094
cg13785123	0.98	0.977	0.977	0.982	0.194	0.84	0.321	0.859
cg14919455	0.955	0.935	0.94	0.968	0.096	0.435	0.093	0.013
cg13079571	0.968	0.971	0.968	0.976	0.164	0.781	0.207	0.26
cg13618516	0.983	0.951	0.966	0.978	0.103	0.405	0.115	0.084
cg23334433	0.977	0.94	0.946	0.966	0.039	0.175	0.056	0.004
cg05171937	0.926	0.98	0.98	0.986	0.14	0.684	0.257	0.655
cg09727050	0.939	0.886	0.92	0.918	0.058	0.265	0.078	0.207
cg12019801	0.971	0.919	0.92	0.971	0.058	0.266	0.091	0.138
cg08279189	0.986	0.959	0.971	0.974	0.048	0.202	0.076	0.004
cg06766034	0.911	0.907	0.876	0.942	0.059	0.367	0.063	0.013
cg05655915	0.946	0.887	0.884	0.907	0.045	0.359	0.059	0.006
cg15891546	0.794	0.806	0.941	0.924	0.084	0.657	0.083	0.005
cg10699171	0.051	0.083	0.07	0.027	0.905	0.613	0.896	0.907
cg01040749	0.973	0.977	0.976	0.977	0.156	0.865	0.284	0.563
cg14268557	0.967	0.955	0.943	0.973	0.084	0.233	0.079	0.025
cg09163720	0.974	0.956	0.96	0.956	0.174	0.719	0.204	0.277
cg21237481	0.989	0.98	0.988	0.979	0.82	0.936	0.735	0.12
cg10342963	0.962	0.961	0.968	0.966	0.282	0.937	0.208	0.021
cg26814100	0.986	0.98	0.982	0.98	0.333	0.818	0.315	0.057
cg18380211	0.974	0.976	0.98	0.976	0.937	0.946	0.844	0.167
cg21346154	0.962	0.987	0.983	0.99	0.588	0.875	0.544	0.118
cg01953317	0.97	0.985	0.983	0.982	0.975	0.977	0.897	0.194
cg23990557	0.968	0.949	0.968	0.957	0.625	0.936	0.549	0.118
cg04128967	0.85	0.916	0.959	0.937	0.854	0.875	0.776	0.127
cg01012879	0.96	0.945	0.967	0.955	0.281	0.94	0.271	0.082
cg01742370	0.969	0.971	0.969	0.977	0.19	0.541	0.198	0.025
cg04003615	0.905	0.908	0.936	0.95	0.322	0.658	0.262	0.025
cg00290355	0.925	0.927	0.949	0.966	0.632	0.78	0.571	0.118
cg17470397	0.993	0.983	0.982	0.992	0.951	0.738	0.842	0.188
cg01655008	0.979	0.962	0.953	0.985	0.268	0.558	0.217	0.047
cg02722672	0.948	0.923	0.951	0.952	0.35	0.815	0.32	0.094
cg04836151	0.986	0.976	0.985	0.99	0.438	0.797	0.411	0.135
cg23012855	0.966	0.97	0.969	0.965	0.9	0.928	0.788	0.21

cg04306507	0.79	0.961	0.961	0.974	0.528	0.762	0.499	0.106
cg25738116	0.949	0.867	0.902	0.887	0.5	0.837	0.457	0.098
cg07769015	0.952	0.926	0.944	0.958	0.259	0.547	0.222	0.04
cg01081737	0.965	0.95	0.946	0.961	0.199	0.508	0.181	0.038
cg26165081	0.965	0.957	0.97	0.974	0.444	0.612	0.407	0.101
cg11690666	0.987	0.976	0.973	0.986	0.19	0.64	0.194	0.082
cg00114012	0.946	0.926	0.918	0.914	0.809	0.923	0.71	0.178
cg06829969	0.831	0.913	0.954	0.965	0.221	0.527	0.175	0.02
cg23540819	0.845	0.88	0.919	0.916	0.484	0.772	0.454	0.092
cg03329755	0.986	0.981	0.987	0.985	0.923	0.976	0.788	0.248
cg25003924	0.866	0.92	0.91	0.934	0.483	0.717	0.445	0.098
cg02832512	0.926	0.853	0.921	0.867	0.184	0.801	0.178	0.057
cg02974085	0.362	0.978	0.979	0.982	0.849	0.847	0.756	0.117
cg02338345	0.736	0.914	0.986	0.98	0.827	0.874	0.746	0.172
cg07268332	0.543	0.931	0.927	0.944	0.144	0.825	0.146	0.021
cg20043649	0.688	0.97	0.975	0.986	0.256	0.499	0.22	0.026
cg22460173	0.828	0.937	0.966	0.942	0.748	0.81	0.653	0.159
cg15586392	0.949	0.925	0.937	0.951	0.184	0.422	0.164	0.029
cg26930596	0.971	0.948	0.942	0.979	0.085	0.307	0.092	0.014

table S1: The blood reference database. This table lists the 333 DHS-DMCs of the blood reference DNAm database, with the DNAm beta-values for each of 7 main blood cell subtypes, as indicated.

CpGs	Genes	Position	Count
cg03636183	F2RL3	chr19:17,000,586	12
cg05575921	AHRR	chr5:373,378	11
cg19859270	GPR15	chr3:98,251,295	10
cg21161138	AHRR	chr5:399,361	9
cg05951221	2q37.1	chr2:233,284,402	8
cg01940273	2q37.1	chr2:233,284,935	7
cg06126421	6p21.33	chr6:30,720,081	7
cg21566642	2q37.1	chr2:233,284,662	7
cg02657160	CPOX	chr3:98,311,063	6
cg03329539	2q37.1	chr2:233,283,329	6
cg06644428	2q37.1	chr2:233,284,113	6
cg14817490	AHRR	chr5:392,920	6
cg25648203	AHRR	chr5:395,445	6
cg26703534	AHRR	chr5:377,358	6
cg03991871	AHRR	chr5:368,448	5
cg09935388	GFI1	chr1:92,947,588	5
cg19572487	RARA	chr17:38,476,025	5

cg23576855	AHRR	chr5:373,300	5
cg24859433	IER3	chr6:30,720,204	5
cg25189904	GNG12	chr1:68,299,493	5
cg01731783	C14orf43	chr14:74,211,789	4
cg01899089	AHRR	chr5:369,969	4
cg04885881	Unknown	chr1:11,123,118	4
cg07123182	KCNQ1	chr11:2,722,391	4
cg08709672	AVPR1B	chr1:206,224,335	4
cg11314684	AKT3	chr1:244,006,289	4
cg11660018	PRSS23	chr11:86,510,915	4
cg12803068	MYO1G	chr7:45,002,919	4
cg12806681	AHRR	chr5:368,395	4
cg12876356	GFI1	chr1:92,946,825	4
cg15342087	IER3	chr6:30,720,210	4
cg20295214	AVPR1B	chr1:206,226,795	4
cg21611682	LRP5	chr11:68,138,269	4
cg22132788	MYO1G	chr7:45,002,487	4
cg27241845	Unknown	chr2:233,250,371	4
cg00310412	SEMA7A	chr15:74,724,919	3
cg01692968	Unknown	chr9:108,005,349	3
cg01901332	ARRB1	chr11:75,031,055	3
cg02451831	KIAA0087	chr7:26,578,099	3
cg03547355	Unknown	chr1:227,003,061	3
cg05284742	ITPK1	chr14:93,552,129	3
cg06060868	SDHA	chr5:231,934	3
cg11207515	CNTNAP2	chr7:146,904,206	3
cg11231349	NOS1AP	chr1:162,050,657	3
cg12075928	PTK2	chr8:141,801,307	3
cg13193840	2q37.1	chr2:233,285,289	3
cg13976502	C14orf43	chr14:74,227,875	3
cg14580211	C5orf62	chr5:150,161,300	3
cg14753356	Unknown	chr6:30,720,109	3
cg18316974	GFI1	chr1:92,947,035	3
cg21121843	HTT	chr4:3,203,983	3
cg21913886	TMEM51	chr1:15,485,346	3
cg22851561	C14orf43	chr14:74,214,183	3
cg23079012	LINC00299	chr2:8,343,711	3
cg23161492	ANPEP	chr15:90,357,203	3
cg23771366	PRSS23	chr11:86,510,999	3
cg23916896	AHRR	chr5:368,805	3
cg24090911	AHRR	chr5:400,732	3
cg24996979	C14orf43	chr14:74,223,355	3
cg25949550	CNTNAP2	chr7:145,814,306	3
cg26271591	NFE2L2	chr2:178,125,956	3

cg26963277	KCNQ1	chr11:2,722,408	3

table S2: Gold-standard list of 62 smoking-associated DMCs (sDMCs). List as curated and defined in Gao et al. Columns label the 450k probe ID, gene symbol, chromosome position and the number of independent whole blood EWAS reporting this probe to be a sDMC.

probeID	t-stat	P	Symbol	Included(ADJ)
cg05575921	-13.04	1E-23	AHRR	TRUE
cg21566642	-12.9	2E-23	NA	TRUE
cg05951221	-12.02	2E-21	NA	TRUE
cg01940273	-11.86	4E-21	NA	TRUE
cg03636183	-10.16	3E-17	F2RL3	TRUE
cg26703534	-9.23	3E-15	AHRR	TRUE
cg21161138	-9.19	4E-15	AHRR	TRUE
cg06126421	-8.48	2E-13	NA	TRUE
cg19859270	-8.33	3E-13	GPR15	TRUE
cg09935388	-8.18	7E-13	GFI1	TRUE
cg03329539	-7.69	8E-12	NA	TRUE
cg15342087	-7.13	1E-10	NA	TRUE
cg26963277	-6.7	0.000000001	NA	TRUE
cg24859433	-6.6	0.000000002	NA	TRUE
cg14817490	-6.56	0.000000002	AHRR	TRUE
cg18316974	-6.55	0.000000002	GFI1	TRUE
cg18146737	-6.45	0.000000004	GFI1	TRUE
cg12876356	-6.39	0.000000005	GFI1	TRUE
cg11660018	-6.29	0.000000008	PRSS23	TRUE
cg25648203	-6.09	0.00000002	AHRR	TRUE
cg00310412	-6.03	0.00000002	SEMA7A	TRUE
cg27241845	-6.01	0.00000003	NA	TRUE
cg20295214	-5.89	0.00000005	AVPR1B	TRUE
cg21322436	-5.8	0.00000007	CNTNAP2	TRUE
cg09662411	-5.47	0.0000003	GFI1	TRUE
cg03991871	-5.4	0.0000004	AHRR	TRUE
cg25949550	-5.39	0.0000004	CNTNAP2	TRUE
cg23771366	-5.22	0.0000009	PRSS23	TRUE
cg23161492	-5.14	0.000001	ANPEP	TRUE
cg12806681	-5.09	0.000002	AHRR	FALSE
cg25189904	-5.08	0.000002	GNG12	TRUE
cg24090911	-5.03	0.000002	AHRR	TRUE
cg08709672	-4.96	0.000003	AVPR1B	TRUE
cg06644428	-4.95	0.000003	NA	TRUE

table S3: Smoking-associated DMCs with no adjustment for cell-type composition. This table lists the 34 smoking-associated DMCs obtained at a FDR < 0.05 in the 152 whole blood Illumina 450k samples from the MRC1946 birth cohort data, only using adjustment for beadchip and no adjustment for changes in blood cell subtype composition. We provide the Illumina probeID, the t-statistic of differential methylation, the associated P-value, the gene symbol (if available) the probe maps to, and whether the probe was included in the top (FDR<0.05) list from the adjusted analysis.

probeID	t-stat	P	Symbol	Included(UnADJ)
cg21566642	-12.45	5E-22	NA	TRUE
cg05575921	-12.43	5E-22	AHRR	TRUE
cg05951221	-12.16	2E-21	NA	TRUE
cg01940273	-11.81	1E-20	NA	TRUE
cg03636183	-9.51	1E-15	F2RL3	TRUE
cg26703534	-8.92	2E-14	AHRR	TRUE
cg21161138	-8.58	1E-13	AHRR	TRUE
cg06126421	-8.43	3E-13	NA	TRUE
cg19859270	-8.4	3E-13	GPR15	TRUE
cg03329539	-7.89	4E-12	NA	TRUE
cg09935388	-7.58	2E-11	GFI1	TRUE
cg15342087	-6.87	5E-10	NA	TRUE
cg24859433	-6.68	0.000000001	NA	TRUE
cg11660018	-6.39	0.000000005	PRSS23	TRUE
cg20295214	-6.37	0.000000006	AVPR1B	TRUE
cg12103569	6.33	0.000000007	SFRS8	FALSE
cg03707168	-6.17	0.00000001	PPP1R15A	FALSE
cg03604011	6.13	0.00000002	AHRR	FALSE
cg14817490	-6.11	0.00000002	AHRR	TRUE
cg18316974	-6.08	0.00000002	GFI1	TRUE
cg06235438	-5.92	0.00000005	ITGAL	FALSE
cg00310412	-5.91	0.00000005	SEMA7A	TRUE
cg18146737	-5.89	0.00000005	GFI1	TRUE
cg14753356	-5.86	0.00000006	NA	FALSE
cg17287155	-5.86	0.00000006	AHRR	FALSE
cg26963277	-5.84	0.00000006	NA	TRUE
cg12876356	-5.77	0.00000009	GFI1	TRUE
cg16771652	-5.64	0.0000002	NOD2	FALSE
cg27241845	-5.55	0.0000002	NA	TRUE
cg25648203	-5.48	0.0000003	AHRR	TRUE
cg21322436	-5.47	0.0000003	CNTNAP2	TRUE
cg24090911	-5.42	0.0000004	AHRR	TRUE
cg04583842	5.41	0.0000004	BANP	FALSE
cg27513574	5.37	0.0000005	SLC34A2	FALSE

cg13470504	5.31	0.0000007	RP3-398D13.1	FALSE
cg04885881	-5.27	0.0000008	NA	FALSE
cg25189904	-5.26	0.0000008	GNG12	TRUE
cg10420527	-5.23	0.0000009	LRP5	FALSE
cg11557636	5.22	0.000001	KIAA1239	FALSE
cg14580211	-5.19	0.000001	MST150	FALSE
cg12147622	-5.19	0.000001	NA	FALSE
cg04332373	5.13	0.000001	CD38	FALSE
cg07826859	-5.08	0.000002	MYO1G	FALSE
cg09662411	-5.07	0.000002	GFI1	TRUE
cg12075928	-5.05	0.000002	PTK2	FALSE
cg25748521	-5.05	0.000002	HIPK2	FALSE
cg06644428	-4.98	0.000003	NA	TRUE
cg06256858	4.94	0.000003	CRMP1	FALSE
cg14114133	-4.93	0.000003	NA	FALSE
cg23161492	-4.93	0.000003	ANPEP	TRUE
cg25949550	-4.92	0.000003	CNTNAP2	TRUE
cg06285727	-4.9	0.000004	ATG16L2	FALSE
cg03843000	4.9	0.000004	TBX5	FALSE
cg23771366	-4.88	0.000004	PRSS23	TRUE
cg03991871	-4.88	0.000004	AHRR	TRUE
cg01763916	-4.88	0.000004	SMAP2	FALSE
cg08047426	4.88	0.000004	ZNF440	FALSE
cg03427663	-4.84	0.000005	NA	FALSE
cg27537125	-4.84	0.000005	NA	FALSE
cg03440944	-4.83	0.000005	C7orf40	FALSE
cg13867915	4.82	0.000005	ARID1A	FALSE
cg14323204	4.81	0.000005	NA	FALSE
cg18625627	4.81	0.000005	TSHR	FALSE
cg08709672	-4.79	0.000006	AVPR1B	TRUE
cg03450842	-4.76	0.000007	ZMIZ1	FALSE
cg23315637	-4.75	0.000007	NA	FALSE
cg07251887	-4.75	0.000007	NA	FALSE
cg02128112	4.72	0.000008	NA	FALSE
cg03907390	4.72	0.000008	GRK5	FALSE
cg05284742	-4.71	0.000008	ITPK1	FALSE

table S4: Smoking-associated DMCs as obtained using EpiDISH. This table lists the 70 smoking-associated DMCs obtained at a FDR < 0.05 in the 152 whole blood Illumina 450k samples from the MRC1946 birth cohort data, after adjustment for changes in blood cell subtype composition (cellular proportions as estimated using EpiDISH) in addition to beadchip. We provide the Illumina probeID, the t-statistic of differential methylation, the associated P-value, the gene symbol (if available) the probe maps to, and whether the probe was included

in the top (FDR<0.05) list from the unadjusted analysis.

References:

1. Reinius LE, Acevedo N, Joerink M, Pershagen G, Dahlen SE, Greco D, Soderhall C, Scheynius A, Kere J: **Differential DNA methylation in purified human blood cells: implications for cell lineage and studies on disease susceptibility.** *PLoS One* 2012, **7:**e41361.
2. Zilbauer M, Rayner TF, Clark C, Coffey AJ, Joyce CJ, Palta P, Palotie A, Lyons PA, Smith KG: **Genome-wide methylation analyses of primary human leukocyte subsets identifies functionally important cell-type-specific hypomethylated regions.** *Blood* 2013, **122:**e52-60.
3. Gao X, Jia M, Zhang Y, Breitling LP, Brenner H: **DNA methylation changes of whole blood cells in response to active smoking exposure in adults: a systematic review of DNA methylation studies.** *Clin Epigenetics* 2015, **7:**113.
